# The Significance of the Myocardial Performance Index and Fetal Doppler Abnormalities in Growth-Restricted Fetuses: A Systematic Review of the Literature

**DOI:** 10.3390/jcm13216469

**Published:** 2024-10-28

**Authors:** Agnieszka Helena Czapska, Katarzyna Kosińska-Kaczyńska

**Affiliations:** Department of Obstetrics, Perinatology and Neonatology, Center of Postgraduate Medical Education, Cegłowska St. 80, 01-809 Warsaw, Poland; ahczapska@gmail.com

**Keywords:** fetal cardiac function, growth-restricted fetuses, myocardial performance index, Doppler assessment, perinatal outcomes, small for gestational age, prenatal cardiac impairment

## Abstract

**Introduction:** This review aims to investigate the clinical implications of using the myocardial performance index (MPI), obtained through tissue Doppler imaging (TDI) and spectral Doppler, in assessing fetal cardiac function in growth-restricted fetuses. It explores the MPI’s potential in predicting adverse perinatal outcomes and its utility when combined with conventional pulsed-wave Doppler assessments for enhanced fetal well-being evaluations. **Material and Methods:** A systematic search of PubMed and Google Scholar databases spanning from 2004 to 2023 was conducted to identify pertinent articles on the MPI’s clinical application in managing growth-restricted fetuses. Inclusion criteria followed the Fetal Medicine Barcelona definition of fetal growth restriction (FGR) to mitigate study group heterogeneity. The research sources were PubMed and Google Scholar databases, and the review was conducted without any specific clinical or laboratory setting. Only articles meeting the inclusion criteria for FGR, as per the Fetal Medicine Barcelona definition, were considered. Six studies meeting these criteria were included in the review. The review analyzed the correlation between MPI values and conventional Doppler parameters, investigating the progression of myocardial function impairment and its association with the risk of fetal demise. The primary outcome measures included the relationship between MPI values, fetal well-being, and the potential for prenatal cardiac dysfunction in growth-restricted fetuses. **Results:** The findings indicate that as conventional Doppler parameters deteriorate, MPI values increase, suggesting progressive myocardial dysfunction. The MPI may cross the 95th percentile before abnormal flow in the ductus venosus and aortic isthmus, highlighting the potential for diastolic dysfunction preceding hypoxia in growth-restricted fetuses. Elevated MPI levels were observed in both growth-restricted and small-for-gestational-age (SGA) fetuses, indicating prenatal cardiac impairment. The strong association between an abnormal MPI and perinatal mortality has been shown for early FGR. **Conclusions:** MPI alterations appear to precede abnormal Doppler parameters in early- and late- onset FGR, potentially indicating diastolic dysfunction preceding hypoxia. Additionally, the MPI correlates with the risk of fetal demise. However, larger studies are needed to establish its sensitivity and specificity. Furthermore, the significance of prenatal cardiac impairment in some SGA fetuses raises questions about its potential impact on perinatal outcomes and cardiovascular programming.

## 1. Introduction

Fetal growth restriction (FGR) arises from various causes, such as maternal conditions (hypertension, preeclampsia), placental insufficiency, infections, or fetal genetic anomalies, and it increases the risk of complications, including cardiac dysfunction. Growth-restricted fetuses face higher risks of reduced cardiac compliance, arterial stiffness, and impaired end-diastolic filling due to long-term oxygen deprivation and pressure overload, leading to cardiac remodeling [1]. FGR requires close monitoring using tools like Doppler ultrasound, cardiotocography (CTG), and biophysical profile scores (BPPs) [2,3], as fetal cardiac impairment often precedes Doppler abnormalities [4]. Despite advances in the diagnostics and management of growth-restricted fetuses, there is still a proportion of pregnancies in which adverse outcomes occur before the Doppler becomes abnormal [5]. This highlights the need for new markers, such as the myocardial performance index (MPI), to better predict adverse outcomes.

**Figure 1:**
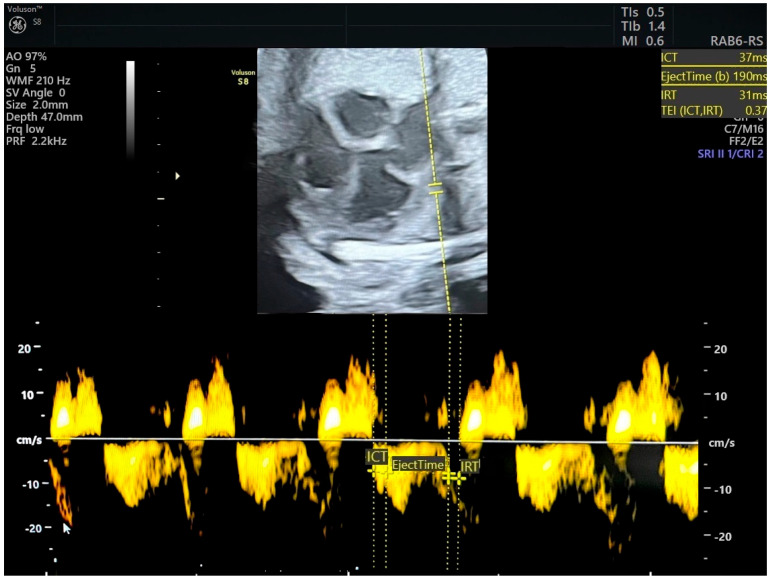
Pulsed tissue Doppler image of tricuspid annulus and measurement of right myocardial performance index. MPI was in normal range (ICT-isovolumetric contraction time, IRT-isovolumetric relaxation time, TEI = MPI). Source of image-personal collection.

**Figure 2:**
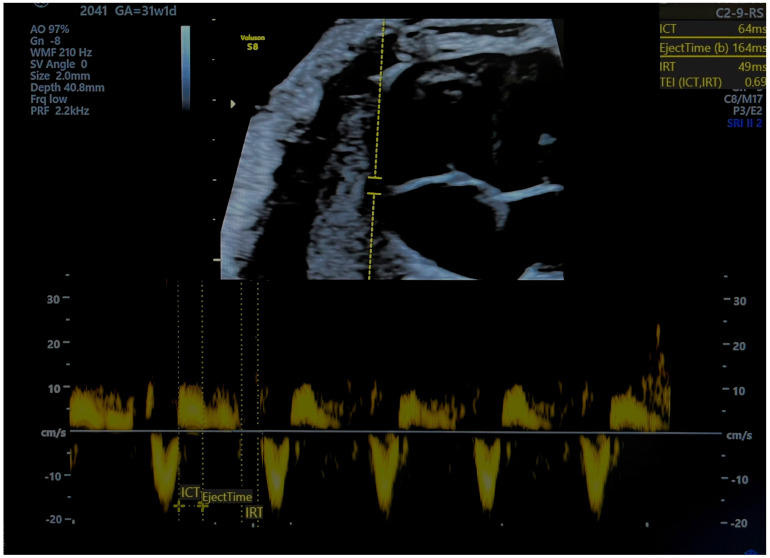
Pulsed tissue Doppler image of tricuspid annulus and measurement of right myocardial performance index. MPI was pathological (ICT-isovolumetric contraction time, IRT-isovolumetric relaxation time, TEI = MPI). Source of image-personal collection.

The MPI is calculated as (ICT + IRT)/ET, representing the isovolumetric contraction, isovolumetric relaxation, and ejection time, respectively [6] (Figure 1 and Figure 2). Independent of fetal cardiac size and geometry [7,8], the MPI increases with the severity of FGR [9,10]. Tissue Doppler imaging (TDI), which measures longitudinal myocardial motion, is more sensitive in detecting early systolic and diastolic dysfunction than conventional Doppler [11]. Combining the MPI with other assessments may improve decision-making on the optimal timing of delivery in FGR cases. The objective of this review is to evaluate the MPI’s efficacy in detecting FGR and predicting adverse neonatal outcomes.

## 2. Methods

A systematic review of the literature, following PRIMSA guidelines, was conducted using the PubMed and Google Scholar databases from 1 May 2023 to 30 May 2023, covering articles published from January 2004 to May 2023. The search strategy aimed to be comprehensive to ensure the inclusion of all relevant studies on the myocardial performance index (MPI) in the management of growth-restricted fetuses (FGR). The search terms included “MPI in FGR”, “TDI in FGR”, and “cardiac function in FGR”, along with variants and synonyms such as “Myocardial Performance Index”, “Tissue Doppler Imaging”, “Fetal Growth Restriction”, “Small for Gestational Age”, “Fetal Cardiac Function”, and “Perinatal Outcomes”. Controlled vocabulary headings were employed for PubMed, including MeSH terms like “Myocardial Performance Index”, “Tissue Doppler Imaging”, “Fetal Growth Retardation”, “Cardiac Function, Fetal”, and “Perinatal Mortality”. Boolean operators were used to combine search terms and refine results, such as (“Myocardial Performance Index” OR “MPI”) AND (“Fetal Growth Restriction” OR “FGR”), and for Google Scholar, search strings included “Myocardial Performance Index” AND “Fetal Growth Restriction” AND “Tissue Doppler Imaging” AND “Cardiac Function” AND “Perinatal Outcomes”. Inclusion criteria were based on the Fetal Medicine Barcelona definition of fetal growth restriction, including estimated fetal weight (EFW) < 10th percentile plus any of the following: EFW < 3rd percentile, cerebroplacental ratio (CPR) < 5th percentile, or uterine artery pulsatility index (UtA PI) > 95th percentile [12]. Articles were excluded if they did not meet the inclusion criteria, were not in English, or were not full-text articles. After removing duplicates, titles and abstracts were screened for relevance, and the full texts of potentially relevant articles were retrieved and assessed for eligibility. Data from the included studies were extracted using a standardized form, and the results were synthesized qualitatively. The synthesis of the studies is presented in the tables provided.

## 3. Results

**Figure 3:**
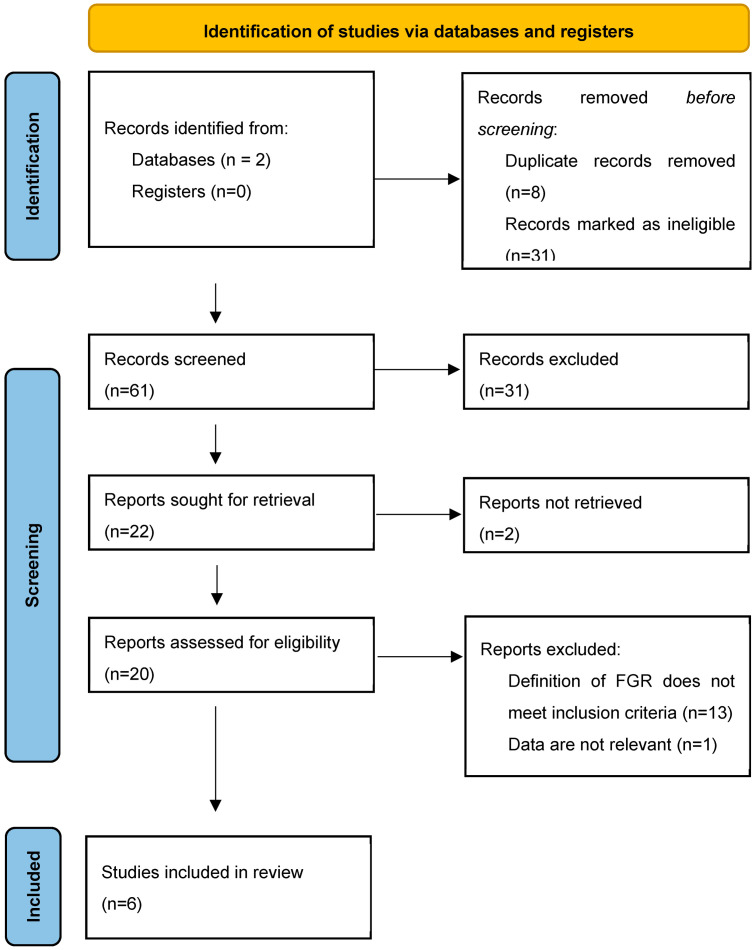
PRISMA 2020 flow diagram for systematic review [13]. *From:* visit: http://www.prisma-statement.org/ accessed on 30 May 2023.

From the total of 61 records found, 22 were sought for retrieval, 20 were assessed for eligibility, and 6 met the inclusion criteria. The process of the identification of studies for this systematic review is presented in the PRISMA flow diagram (Figure 3). The studies included in the review are summarized in Table 1.

## 4. Discussion

### 4.1. Main Findings

This is the first systematic review of the literature encompassing MPI’s significance in the management of FGR whose inclusion criteria are based on a unified definition of FGR. The authors hypothesized that the integration of MPI in standard clinical assessments could improve the accuracy of detecting the deterioration in FGR fetuses’ well-being and therefore help to predict adverse perinatal outcomes, leading to more comprehensive decisions regarding the optimal timing of delivery. In accordance with our hypothesis, we found that the MPI becomes altered before the venous and arterial Doppler indices, giving the possibility of identifying fetuses at early stages of cardiac function impairment. Moreover, the MPI increases with the severity of growth restriction and is associated with a higher risk of fetal demise. What is more, there is a group of fetuses classified as SGA, who present higher MPI values in comparison to appropriate-for-gestational-age (AGA) fetuses, which may suggest that instead of being “constitutionally small” they suffer true growth restriction.

### 4.2. Interprettion

#### 4.2.1. Early-Onset FGR

Early-onset FGR is a serious condition with a multifactorial background. In the latest work of Palalioglu et al. 30 early-onset FGR fetuses were included as a study group and matched with 46 AGA fetuses, all between 24 and 34 weeks of gestation [11]. The authors used the TDI technique and proved that MPI, ICT, and IRT increase in growth-restricted fetuses who have either a normal or abnormal UA Doppler, whereas both study and control groups showed no statistically significant difference according to the DV PI and DV PI percentile [11]. This finding supports the view that diastolic dysfunction and an increase in MPI may occur prior to hypoxia in FGR fetuses. The authors proved that MPI values vary throughout pregnancy and propose the MPI cut-off value to be set at 0.47 [11]. An increase in MPI above this level showed a 97.83% sensitivity and 86.67% specificity in detecting FGR [11]. The work of Cruz-Martinez et al. supports the aforementioned results regarding the sequence of changes in the Doppler parameters [10]. The authors incorporated 115 early-onset FGR fetuses with a UA PI > 95th percentile and performed a serial ultrasound examinations every 1–7 days, measuring the MPI, AoI PI, and DV PI [10]. All fetuses were delivered before 34 weeks of gestation; indications for delivery were DV abnormalities, CTG decelerations, an abnormal BPP, UA AEDF, or UA REDF, maternal complications due to preeclampsia, or fetal demise [10]. In early-onset FGR, all Doppler indices exhibited a continuous decline, but the pace of this deterioration varied [10]. The MPI crossed the 95th percentile 26 days before delivery, whereas the AoI PI and DV PI flow crossed the 95th percentile 23 and 5 days before delivery, respectively [10].

Data about the MPI’s significance in predicting adverse perinatal outcomes remain conflicting. In 2009, Hernandez-Andrade et al. investigated the MPI and AoI flow as independent parameters and in correlation with the UA PI, middle cerebral artery PI (MCA PI), and DV PI in terms of the prediction of fetal demise [16]. Only Doppler and MPI results obtained 72 h prior to delivery or fetal death were taken into account. An abnormal MPI was defined as above the 95th percentile and an abnormal DV flow was defined as absent or reversed. The MPI and DV occurred to be independent predictors of fetal death, and combining both parameters increased the predictive accuracy [16]. In FGR fetuses below 28 weeks of gestation, the probability of mortality was 18% when the fetus displayed normal atrial flow in the DV and a normal MPI. When either of these characteristics was abnormal, the risk of death increased to 70–73%. If both the DV and MPI were abnormal, the likelihood of mortality rose significantly to 97%. For FGR fetuses beyond 28 weeks of gestation, the risk of death was minimal (0.1%) when the DV and MPI were normal. However, if either of these parameters were abnormal, the probability of mortality increased to 6–7%. In cases where both atrial flow in the DV and the MPI were abnormal, the risk of death reached 45% [16]. In 2012, Cruz-Lemini et al. presented work that did not support the previous findings of Hernandez-Andrade et al. [13,15,16]. According to this research, despite the MPI’s association with perinatal demise, it has no additive value in comparison to DV assessment [15]. This inconsistency may be due to the statistical approach and the limited external validity of the MPI. Furthermore, the MPI becomes altered earlier than the DV, and this fact may also explain its weaker association with perinatal mortality. It is worth outlining that the FGR criteria were the same in both studies (EFW < 10th percentile and UA PI > 95th percentile), the gestational age (GA) was below 34 weeks, and the MPI was measured using the spectral Doppler technique.

Another study evaluating the correlation between MPI values and adverse perinatal outcomes is the work of Bhorat et al. [17]. The authors included in the study 43 FGR fetuses between 28 and 34 weeks of gestation described as having an abdominal circumference (AC) below the 10th percentile for GA and an abnormal umbilical artery resistance index (UA RI) >2 standard deviations [17]. This definition of FGR does not meet the inclusion criteria of this review as outlined by the FGR definition of Fetal Medicine Barcelona. However, the definition of FGR incorporated by Bhorat et al. is close to the criteria of the Delphi consensus (early-onset FGR: GA < 32 weeks is defined by AC/EFW < 3rd percentile or UA AEDF or AC/EFW < 10th percentile combined with UtA PI > 95th percentile and/or UA PI > 95th percentile; late-onset FGR: GA ≥ 32 weeks is defined by AC/EFW < 3rd percentile or two out of three of the: AC/EFW < 10th percentile or AC/EFW crossing centiles >2 or CPR < 5th percentile/UA PI > 95th percentile) [17,18]. Adverse perinatal outcomes were defined as perinatal death, neonatal resuscitation, hypoxic–ischemic encephalopathy, neonatal pH < 7.15, intraventricular hemorrhage, and bronchopulmonary dysplasia [17]. The MPI was assessed with a spectral Doppler, and the left myocardial performance index (LMPI) was obtained. The authors reported that MPI values increased with the deterioration in Doppler parameters in early-onset FGR fetuses [17]. This finding is in line with the aforementioned works of Palalioglu et al. and Cruz-Martinez et al. [10,11]. MPI cut-off values were defined as 0.59 for early-onset FGR and 0.37 for AGA controls [16]. An MPI of 0.54 had a sensitivity of 87%, a specificity of 75%, and a likelihood ratio of 3.47 for an adverse perinatal outcome occurrence [16]. A cut-off MPI value of 0.67 conferred a sensitivity of 100%, a specificity of 81%, and a likelihood ratio of 5.28 for perinatal death [16]. The bias of this work is the FGR fetus selection criteria and small study group.

#### 4.2.2. Late-Onset FGR

There are not much data considering MPI’s incorporation in the management of late-onset FGR. Kaya et al. studied 40 SGA and 40 late-onset FGR fetuses between 34 and 37 weeks of gestation and demonstrated that MPI changes occur prior to abnormal precordial venous flow [14]. Moreover, cardiac function impairment was observed also in some SGA fetuses, which can be an alarming sign of possible adverse perinatal outcomes in the group classified according to spectral Doppler as constitutionally small [14]. MPI values in healthy controls were 0.45 ± 0.06 [14]. In comparison to the controls, MPI results obtained in SGA fetuses 0.63 ± 0.13 and in late-onset FGR fetuses 0.69 ± 0.14 were statistically significant (*p* < 0.001) [14]. These findings also support the work of Perez-Cruz et al., where the authors studied 150 late-onset FGR and 59 SGA fetuses matched with 150 healthy AGA controls and found signs of prenatal cardiac dysfunction in both study groups [9]. A conventional Doppler and TDI were used to measure the right myocardial performance index (RMPI) and LMPI; the septal MPI was measured only by means of TDI. Nearly all MPI measurements, both obtained by means of TDI and conventional Doppler, in study groups of small fetuses (SGA and FGR) compared to controls (AGA) were statistically significant (*p* < 0.05) [9]. Only the RMPI measurement in SGA fetuses 0.57 ± 0.18 versus AGA controls 0.51 ± 0.12 obtained by conventional Doppler was not statistically significant (*p* > 0.05) [9]. RMPI values measured with TDI in AGA fetuses were 0.50 ± 0.09; in the study groups, the results were statistically significant versus AGA controls: SGA fetuses 0.56 ± 0.13 (*p* < 0.05) and late-onset FGR fetuses 0.58 ± 0.11 (*p* < 0.05) [9]. LMPI values measured with TDI in AGA fetuses were 0.51 ± 0.09; in the study groups, the results were statistically significant versus AGA controls: SGA fetuses 0.56 ± 0.11 (*p* < 0.05) and late-onset FGR fetuses 0.62 ± 0.11 (*p* < 0.05) [9]. Septal MPI values measured with TDI in AGA fetuses were 0.52 ± 0.08; in the study groups, the results were statistically significant versus AGA controls: SGA fetuses 0.6 ± 0.11 (*p* < 0.05) and late-onset FGR fetuses 0.6 ± 0.10 (*p* < 0.05) [9]. Additionally, in comparison to the control group, FGR and some SGA fetuses presented cardiac shape changes and a decrease in the longitudinal motion of the myocardium [9]. It is essential to note that some fetuses classified as constitutionally small according to pulse Doppler parameters suffer true growth restriction and are at risk of perinatal cardiovascular programming [9].

### 4.3. Strengths and Limitations

The bias of this review is the fact that some of the studies included small study groups [11,16]. Moreover, there is a discrepancy in the methodology of MPI used. Newer studies published after 2015 [9,11,14] used TDI to calculate the MPI, whereas older works [10,15,16] used spectral Doppler to obtain MPI measurements. The methodology of spectral Doppler MPI measurement consists of obtaining an apical five-chamber view; then, a gated pulsed Doppler sample volume is positioned in the left ventricle at the junction of the anterior mitral valve leaflet and the left ventricular outflow tract [19]. The following time intervals are recorded: the interval from the end of the A-wave (which represents late diastolic filling of the left ventricle, which occurs during atrial contraction) to the beginning of the aortic pulsed Doppler tracing (ICT), the duration from the start to the end of the aortic pulsed Doppler tracing (ET), and the interval from the end of the ejection time to the beginning of the E-wave (which represents early diastolic filling of the left ventricle) (IRT) [20]. TDI is believed to be a more sensitive technique in the evaluation of cardiac impairment in growth-restricted fetuses, as it permits a quantitative assessment of the changes in the longitudinal motility of the myocardium. Moreover, TDI is less dependent on preload, afterload, and fetal heart rate than conventional Doppler [11,14]. Also, the pathophysiologic changes in the FGR circulation, namely increased right ventricle afterload resulting from increased systemic and pulmonary vascular resistance and decreased left ventricle afterload resulting from cerebral vasodilatation, induce changes in cardiac output and therefore reduce the reliability of spectral Doppler measurements [11]. Another bias encompasses the type of MPI used too; some authors used the LMPI [10,15,16], others preferred the RMPI [11,14] and one author used both the LMPI and RMPI [9]. The RMPI is believed to be more sensitive, as the right chamber is dominant in the fetal life and changes in right heart function precede the impairment of left heart function [11,14]. It is worth outlining that early FGR has been very well known to be associated with a higher risk of perinatal mortality due to the early gestational age at delivery, very low birthweight, and increased respiratory perinatal morbidity. For these reasons, another important bias that should be highlighted is that in this specific subset of pregnancies, the risk of mortality can be related to all these factors and not only to cardiac impairment.

This review supports the hypothesis that the MPI can contribute to a more comprehensive approach to the treatment and management of growth-restricted fetuses. Nearly all studies whose inclusion criteria for the study group were in line with the current Fetal Medicine Barcelona definition of FGR showed the potential advantage of incorporating the MPI in clinical practice. TDI can detect cardiac dysfunction at a very early stage and is more reproducible and sensitive than conventional Doppler. As a result, MPI obtained by means of TDI may be helpful in selecting the FGR group which needs closer monitoring and more careful postnatal follow-up. Larger, multicenter studies are needed to define the MPI cut-off values and assess the potential contribution of the MPI in the algorithm of the monitoring and management of FGR fetuses. It is worth outlining how crucial in future studies will be the proper selection of the FGR study group, the implementation of the TDI technique, and the use of RMPI measurement.

FGR induces pathophysiological changes to which primarily the fetal heart adapts in a chronic subclinical way. Firstly, the diastolic function of the heart and its compliance, that is the relaxation capacity, become altered [20]. These subtle changes can be seen in myocardium longitudinal movement alterations, as longitudinal fibers in comparison to radial ones are the furthest from the coronary circulation and the most susceptible to pressure overload [20]. By the mode of functional echocardiography, we can assess myocardial deformity and myocardial motility, that is myocardial motion (MAPSE and TAPSE parameters) and the myocardial velocity at which the muscle moves (the tissue Doppler assessment of systolic waves and biphasic diastolic waves) [20]. As well as measuring the peak systolic and diastolic velocities of the myocardium, we can also measure ET, ICT, and IRT and calculate the MPI. From the pathophysiological point of view, firstly there will be an alteration in the muscle function before a change in the blood flow [20]. Therefore, an MPI measured by TDI should be altered earlier than an MPI measured by conventional pulse Doppler. The MPI’s significance in early-onset FGR and late-onset FGR is summarized in Table 2. Recent works suggest that the MPI measured by TDI increases in early-onset FGR fetuses before the umbilical artery Doppler becomes abnormal [11]. As far as late-onset FGR is concerned, the authors report that a proportion of fetuses classified as SGA have increased MPI values; therefore, these fetuses may suffer true growth restriction, and the MPI becomes altered before changes in the blood flow reach cut-off values for FGR [21,22]. Fetal myocardial performance deteriorates in concordance with the severity of growth restriction; however, at the same time, it increases throughout the gestation also in AGA fetuses [22,23]. As for FGR fetuses, we can observe the following parameters in the presented order crossing the 95 percentile: umbilical artery pulsatility index (UA PI), aortic isthmus pulsatility index (AoI PI), and ductus venous pulsatility index (DV PI) [10,12]. Abnormal AoI flow precedes DV abnormalities by around 1 week [10,12]. In a similar period of time, umbilical artery flow may become absent or reversed (UA AEDF or UA REDF), which also happens around 1 week before acute deterioration [12]. These changes indicate advanced hypoxia and acidosis. The last phase is fetal heart systolic dysfunction, myocardial radial fibers’ movement impairment, alterations in the ejection fraction and cardiac output, reversed arterial flow in the DV, computed cardiotocography short time variation < 3 ms (cCTG STV), and abnormal BPP and CTG decelerations, all leading to serious fetal injury or death [12,14].

## 5. Conclusions

In conclusion, this study elucidates temporal and causal relationships in the cardiovascular dynamics in FGR, providing valuable insights into prenatal cardiac adaptations and their potential clinical implications. The MPI becomes altered before DV and Ao flow, which means that diastolic dysfunction occurs prior to hypoxia in both early- and late-onset FGR fetuses. The documented association between the MPI value’s increase and the deterioration of Doppler parameters in both early- and late-onset FGR underscores the MPI as an early indicator of fetal cardiac compromise. The MPI shows a correlation with the risk of fetal demise; however, the clinical utility of the MPI in predicting adverse perinatal outcomes necessitates rigorous assessment through expansive, multi-center studies to ascertain its sensitivity, specificity, and predictive capacity.

In extending our analysis to SGA, it is noted that prenatal cardiac impairment may occur also in this subgroup. The meaning of this finding and its association with adverse perinatal outcomes, as well as perinatal cardiovascular programming, needs further investigation. A comprehensive exploration of these aspects has the potential to advance our comprehension of fetal cardiovascular health and its enduring repercussions.

In synthesis, this study advances our understanding of MPI dynamics, their alignment with Doppler parameters in FGR, and their predictive associations with adverse perinatal outcomes. Furthermore, it emphasizes the concept of fetal cardiac impairment in SGA fetuses, prompting further investigation. These findings collectively highlight the urgency of robust research to validate and extend these observations.

## Figures and Tables

**Table 1:** Synthesis of the studies included in the review.

Reference	Group Studied	Gestational Age	Inclusion Criteria	Exclusion Criteria	MPI Used	Pulse Doppler Parameters Studied	Perinatal Outcomes Studied	Main Findings
Palalioglu et al. (2021) [11]	EO-FGR (30) controls (46)	24–34 weeks	EFW < 3rd pc, or EFW < 10th pc with UA PI > 95th pc and/or CPR < 5th pc and/or mean UtA-PI > 95th pc	multiple pregnancies, fetal structural or chromosomal anomaly, diabetes, cholestasis, preeclampsia, FGR diagnosis below the viability limit, early membrane rupture, oligohydramnios, fetal heart complication, or any maternal medical illness	TDI/RMPI	UA PI, UA PI pc, MCA PI, MCA PI pc, CPR, CPR pc, DV PI, DV PI pc, UtA PI, UtA PI pc	GA at delivery, birth weight, 1 min Apgar score, 5 min Apgar score, umbilical artery pH, duration of NICU hospitalization	MPI values vary slightly throughout the pregnancy and have an average of 0.36 MPI (between 0.28–0.44). Normal MPI cut-off value was calculated as 0.47 and below. With an MPI above 0.47, the sensitivity and specificity for detecting FGR were 97.83% and 86.67%, accordingly. MPI increases in FGR fetuses with a normal umbilical artery Doppler, which may be an early marker of hypoxia in the mechanism of diastolic impairment.
Kaya et al. (2019) [14]	LO-FGR (40) SGA (40) controls (40)	34–37 weeks	EFW < 3rd pc, or EFW < 10th pc with UA PI > 95th pc and/or cerebroplacental ratio (CPR) < 5th pc and/or mean UtA-PI > 95th pc. SGA was defined as an EFW between 3rd and 9th pc with normal Doppler indices.	multiple pregnancies, fetal structural or chromosomal anomaly, fetal infection, presence of fetal arrhythmia or non-reassuring fetal heart rate pattern, presence of maternal comorbidities (chronic hypertension, preeclampsia, diabetes mellitus, chronic renal disease), maternal tobacco use	TDI/RMPI	UA PI, MCA PI, CPR, DV PI, UtA PI	interval between diagnosis and delivery, GA at delivery, birth weight, 1 min Apgar score, 5 min Apgar score, umbilical artery pH, NICU stay >14 days	Prenatal cardiac impairment was observed both in SGA and LO-FGR fetuses. MPI measurements: AGA 0.45 ± 0.06, SGA 0.63 ± 0.13, LO-FGR 0.69 ± 0.14. There were significantly increased levels of MPI values in SGA and LO-FGR fetuses compared to the control group (*p* < 0.001).
Perez-Cruz et al. (2015) [9]	LO-FGR (150) SGA (59) controls (150)	Delivery >34 weeks	EFW < 3rd pc, or EFW < 10th pc with cerebroplacental ratio (CPR) < 5th pc and/or mean UtA-PI > 95th pc. SGA was defined as an EFW between 3rd and 9th pc with normal Doppler indices.	fetal structural or chromosomal anomalies, fetal infection, gestational diabetes, multiple pregnancy, IVF, or ICSI pregnancies	TDI and SD/RMPI, LMPI, septal MPI	UA PI, MCA PI, CPR, DV PI, UtA PI	GA at delivery, birth weight, birth weight percentile, 5 min Apgar score < 8, umbilical artery pH, cord blood glucose, NICU stay >14 days, morbidity *, mortality	Both LO-FGR and SGA fetuses showed larger and more globular hearts compared to controls. Both LO-FGR and SGA fetuses presented cardiac dysfunction with higher LMPI measurements: AGA 0.45 ± 0.14, SGA 0.51 ± 0.08, LO-FGR 0.57 ± 0.1. Large proportion of SGA fetus have true growth restriction and suffer fetal cardiovascular programming.
Cruz-Lemini et al. (2012) [15]	EO-FGR (157)	delivery or death <34 weeks	EFW < 10th and UA-PI > 95th pc	twin pregnancies, fetal structural or chromosomal abnormalities, birth weight >10th percentile, fetal infection	SD/LMPI	UA PI, MCA PI, DV PI, AoI IFI	GA at delivery, birth weight, 5 min Apgar score, umbilical cord arterial pH	MPI do not improve the predictive value of GA and DV in terms of perinatal mortality in EO-FGR fetuses. GA has the strongest predictive value in terms of perinatal mortality in EO-FGR below 26 weeks. DV flow allows the stratification of risk of perinatal death in EO-FGR fetuses delivered between 26 and 28 weeks of gestation.
Cruz-Martinez et al. (2011) [10]	EO-FGR (115)	Delivery <34 weeks	EFW < 10th and UA-PI > 95th pc	congenital malformations and chromosomal abnormalities, birth weight >10th percentile	SD/LMPI	UA PI, DV PI, AoI PI	not applicable	In EO-FGR, the MPI becomes altered before AoI and DV; these parameters crossed the 95th percentile 26, 12, and 5 days before delivery, respectively.
Hernandez-Andrade et al. (2009) [8]	EO-FGR (97)	delivery or death between 24 and 34 weeks	EFW < 10th and UA-PI > 95th pc	fetal structural or chromosomal abnormalities, fetal infection, birth weight >10th percentile	SD/LMPI	UA PI MCA PI DV PI AoI IFI	GA at delivery, birth weight, birth weight percentile, 5 min Apgar score <7, umbilical artery pH < 7.2, days in NICU, perinatal death, bronchopulmonary dysplasia, intraventricular hemorrhage III–IV, necrotizing enterocolitis, adverse outcome	DV PI and MPI are independent predictors of perinatal death in EO-FGR. Combining DV PI and MPI has a better predictive value for perinatal death in EO-FGR than any single parameter.

* perinatal death, bronchopulmonary dysplasia, hyaline membrane disease, intraventricular hemorrhage grade 3 or 4, necrotizing enterocolitis, sepsis, retinopathy grade 3 or 4. EO-FGR—early-onset fetal growth restriction, LO-FGR—late-onset fetal growth restriction, SGA—small for gestational age, AGA—appropriate for gestational age, EFW—estimated fetal weight, pc—percentile, UA PI—umbilical artery PI, MCA PI—middle cerebral artery PI, CPR—cerebroplacental ratio, DV PI—ductus venous PI, UtA PI—uterine artery PI, AoI PI—aortic isthmus PI, AoI IFI—aortic isthmus blood flow velocity index, MPI—myocardial performance index, RMPI—right myocardial performance index, LMPI—left myocardial performance index, TDI—tissue Doppler imaging, SD—spectral Doppler, NICU—neonatal intensive care unit.

**Table 2:** MPI significance in early- and late-onset FGR.

Early-Onset FGR	Late-Onset FGR
RMPI TDI Values: Average 0.36 (0.28–0.44). Normal RMPI cut-off: 0.47 and below.	RMPI TDI Values: - Kaya et al. (2019) [14]: - AGA: 0.45 ± 0.06 - SGA: 0.63 ± 0.13 - LO-FGR: 0.69 ± 0.14 Perez-Cruz et al. (2015) [9]: - AGA: 0.50 ± 0.09 - SGA: 0.56 ± 0.13 - LO-FGR: 0.58 ± 0.11
Timing: MPI alters before AoI and DV by 26, 12, and 5 days before delivery, respectively, which means that diastolic dysfunction occur prior to hypoxia.	Timing: MPI becomes altered before AoI and DV flow, which means that diastolic dysfunction occurs prior to hypoxia.
Cardiac Impairment: - MPI increases in EO-FGR fetuses with normal umbilical artery Doppler, indicating early hypoxia and diastolic impairment. - The documented link between increases in MPI values and the worsening of Doppler parameters highlights MPI as an early indicator of fetal cardiac compromise.	Cardiac Impairment: - Observed in both SGA and LO-FGR fetuses. Both groups have larger, more globular hearts. - The documented link between increases in MPI values and the worsening of Doppler parameters highlights MPI as an early indicator of fetal cardiac compromise.
Predictive Value: - MPI does not improve predictive value of GA and DV for perinatal mortality; GA is the strongest predictor below 26 weeks. - RMPI TDI > 0.47 has 97.83% sensitivity and 86.67% specificity for detecting EO-FGR.	Predictive Value: A proportion of fetuses classified as SGA have increased MPI values; therefore, these fetuses may suffer true growth restriction, and the MPI becomes altered before changes in the blood flow reach cut-off values for FGR.
